# β-Glucan exacerbates allergic airway responses to house dust mite allergen

**DOI:** 10.1186/s12931-016-0352-5

**Published:** 2016-04-02

**Authors:** Sabelo Hadebe, Frank Kirstein, Kaat Fierens, Pierre Redelinghuys, Graeme I. Murray, David L. Williams, Bart N. Lambrecht, Frank Brombacher, Gordon D. Brown

**Affiliations:** Aberdeen Fungal Group, Infection, Immunity and Inflammation Programme, University of Aberdeen, Aberdeen, UK; International Centre for Genetic Engineering and Biotechnology and Division of Immunology, Institute of Infectious Disease and Molecular Medicine, Faculty of Health Science, University of Cape Town, Cape Town, South Africa; VIB Inflammation Research Center, Laboratory of Immunoregulation and Mucosal Immunology, University Ghent, Ghent, 9000 Belgium; Pathology, Division of Applied Medicine, Institute of Medical Sciences, Foresterhill, University of Aberdeen, Aberdeen, AB25 2ZD UK; Department of Surgery and Center for Inflammation, Infectious Disease and Immunity, James H. Quillen College of Medicine, East Tennessee State University, Johnson City, TN USA; Department of Pulmonary Medicine, Erasmus MC, Rotterdam, The Netherlands; Aberdeen Fungal Group, MRC Centre for Medical Mycology, Infection, Immunity and Inflammation Programme, School of Medicine & Dentistry, Institute of Medical Sciences, University of Aberdeen, Foresterhill, Aberdeen, AB25 2ZD UK

**Keywords:** β-glucans, Allergy, Eosinophil, T helper 2, House dust mite

## Abstract

β-(1,3)-Glucan is present in mould cell walls and frequently detected in house dust mite (HDM) faeces. β-Glucan exposure is thought to be associated with pulmonary allergic inflammation in mouse and man, although the published data are inconsistent. Here, we show that highly purified β-glucan exacerbates HDM-induced eosinophilic, T helper 2 type airway responses by acting as an adjuvant, promoting activation, proliferation and polarisation of HDM-specific T cells (1-Derβ T cells). We therefore provide definitive evidence that β-glucan can influence allergic pulmonary inflammation.

## Results

Asthma is a common chronic obstructive airway disease, which presents as episodes of wheeze, shortness of breath and chest tightness, and in extreme cases the disease can be fatal [1, 2]. It is traditionally a disease of the developed world, with increasing incidence both in childhood and adulthood [3]. Asthma is widely regarded as a T helper 2 (Th2) cell-mediated disease, although other forms exist [2]. Th2 type asthma can be characterised by eosinophil accumulation in the alveolar space and cytokines, including interleukin (IL-) IL-4, IL-5 and IL-13, as well as by other physiological changes such as goblet cell hyperplasia [1]. The underlying factors contributing to the disease are numerous and not well understood. Environmental allergen sensitisation is known to play a major part in asthma development and exacerbation. Fungal spores are one of many environmental allergens encountered daily and their exposure directly correlates with increased incidence of asthma episodes and hospital admission [4]. β-(1,3)-Glucan (β-glucan) is a pathogen-associated molecular pattern (PAMP) mainly present in fungal cell walls, but also present in bacteria, plants and has been detected in house dust mite (HDM) faeces [5]. β-Glucan has been implicated in both innate and allergic respiratory inflammatory responses, however, studies in both human and animal models are inconsistent [6]. These discrepancies are due, in large part, to the purity and solubility of the β-glucan preparations used [6, 7].

**Fig. 1:**
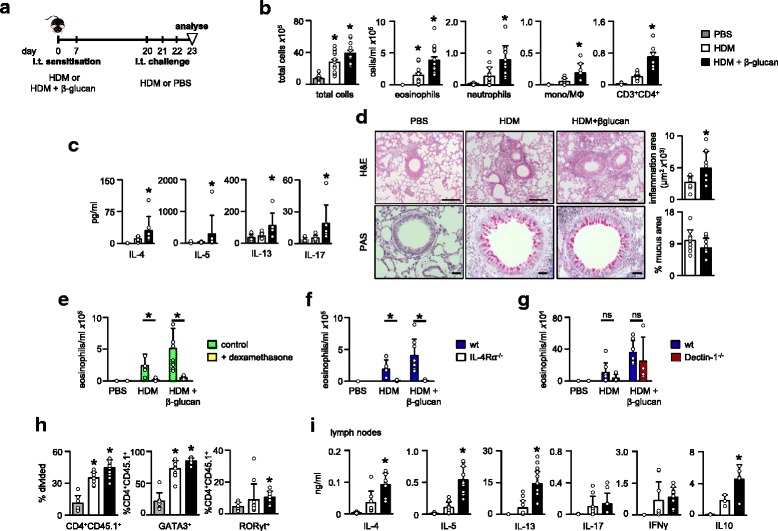
β-Glucans promote Th2 allergic airway inflammation to HDM allergen both during sensitisation and challenge stages through IL-4Rα. **a** Timeline for HDM sensitisation and challenge of C57BL/6 mice. Mice were sensitised i.t. with HDM alone (10 μg, Greer Laboratories, Lenoir, NC, *Dermatophagoides pteronyssinus* (Der p1) 145.56 mcg per vial, endotoxin 31.25 EU per vial, 2.87 mg protein per vial and 11.6 mg dry weight per vial) or together with β-glucan (1×10^7^ particles ≈ 10 μg), highly purified from *Saccharomyces cereviseae* [8] at day 0 and 7 and challenged i.t. with HDM (10 μg) alone at day 20, 21 and 22. Control mice were sensitised and challenged with PBS at the same time-points. **b** Number of total leukocytes, eosinophils (Siglec-F^hi^Gr-1^lo^CD11c^lo^), neutrophils (Gr-1^hi^CD11b^hi^F4/80^lo^), monocytes/macrophages (F4/80^hi^Gr-1^lo^CD11b^hi^) and T-cells (CD3^+^CD4^+^) in the BALF of mice at day 23. **c** Cytokine concentrations in BALF were detected by Bio-Plex Pro Mouse cytokine 23-plex Assay (Bio-Rad Laboratories Ltd, USA), according to the manufacturer’s specifications. **d** Haematoxylin and eosin **h** & **e**) and Periodic Acid Schiff (PAS) stained lung sections, wax embedded and formalin fixed. Bar charts (right) show quantification of the inflammation and of mucus producing goblet cells in the H&E and PAS stained sections, respectively. Scale bars represent 100 μm (*H&E*) and 50 μm (*PAS*). **e** Mice were sensitised and challenged as in (**a**), except one group of animals received dexamethasone 21-phosphate disodium salt (Sigma-Aldrich, St. Louis, MO, USA) i.p. (3 mg/kg in 100 μl) on days 20, 21 and 22. **f** Number of eosinophils (Siglec-F^hi^Gr-1^lo^CD11c^lo^) in the BALF of wild type and IL-4Rα^−/−^ mice. **g** Number of eosinophils (Siglec-F^hi^Gr-1^lo^CD11c^lo^) in the BALF of wild type and Dectin-1^−/−^ mice. **h** Proliferation (CFSE dilution frequency) or GATA3 expression (CD45.1^+^CD3^+^CD4^+^CD44^hi^GATA3^+^) or RORγt expression (CD45.1^+^CD3^+^CD4^+^CD44^hi^RORγt^+^) in adoptively transferred 1-Derβ specific T cells (CD45.1^+^CD45.2^−^CD3^+^CD4^+^CD44^hi^CFSE^+^) in the MLN of recipient mice three days after sensitisation. For adoptive transfer, 1-Derβ TCR T cells were isolated from spleen and MLNs of naïve 1-Derβ TCR transgenic mouse, stained with CFSE and then transferred (1×10^7^ cells/mouse) to WT C57BL/6 mice 2 h before sensitisation (i.t.) with HDM alone or together with β-glucan. **i** Cytokine production by MLN cell suspensions isolated from mice three days after sensitisation, as above, restimulated ex vivo with HDM (15 μg) for 3 days (cytokines were detected by ELISA (eBiosciences), according to manufacturer’s instructions). Shown are the mean ± SD of pooled data from at least two independently repeated experiments. *, *p* < 0.05, ns, not significant

Here, we made use of highly purified particulate β-glucans of similar size to fungal spores, but without contaminating agonists [8] and have investigated their effects on pulmonary inflammation in the context of HDM-induced responses. For these experiments, we sensitised C57BL/6 mice intratracheally (i.t) with HDM alone or with HDM together with β-glucan and subsequently challenged these mice i.t. with HDM only or PBS as a control (Fig. 1a). Mice sensitised and challenged with HDM alone developed eosinophilic pulmonary inflammation in the bronchoalveolar lavage fluid (BALF) (Fig. 1b), as previously shown [9]. However, when HDM plus β-glucan sensitised mice were challenged with HDM, they developed a more profound pulmonary inflammation, characterised by significantly higher numbers of eosinophils (Fig. 1b). There were also slight, but significant, increases in the numbers of neutrophils, monocytes/macrophages and T-cells (Fig. 1b; note the difference in scales). Consistent with these observations, higher levels of IL-4, IL-5, IL-13 and IL-17 were detected in the BALF of mice sensitised with HDM plus β-glucan (Fig. 1c). Moreover, we also observed increased inflammation by histology in these mice, but not in mucus-producing goblet cells (Fig. 1d). Similar effects on Th2-type inflammatory responses were obtained when mice were sensitised with HDM alone and then challenged with HDM together with β-glucan (data not shown). Unlike our previous observations following co-administration of β-glucan plus lipopolysaccharide (LPS) [10], sensitisation with HDM in the presence of β-glucan alone did not induce steroid (dexamethasone) resistant responses (Fig. 1e). Thus these results demonstrate that β-glucan can influence the development of Th2-mediated allergic inflammatory responses during sensitisation and challenge.

We next explored the mechanisms underlying the effects of β-glucan on allergic responses. We first determined if IL-4 receptor α (IL-4Rα) was essentially required for the β-glucan-mediated effects observed in our model [11]. Indeed, loss of IL-4Rα completely abrogated the eosinophilic airway inflammation in the presence of β-glucans (Fig. 1f). We then determined if the exacerbated responses induced by β-glucan were being mediated by the major beta-glucan receptor, Dectin-1 [12]. Unexpectedly, we found that loss of this receptor had no significant effect on the enhanced eosinophilic response induced by β-glucans (Fig. 1g). This suggests that other systems are mediating these activities, or compensating for the loss of Dectin-1, such as CR3 and/or complement [7, 13].

To gain further insights, we next explored allergic T-cell responses by making use of a T cell receptor (TCR) transgenic (Tg) mouse that recognises an immuno-dominant peptide from the HDM-derived allergen, Derp-1 (1-Derβ Tg) [14]. We found that adoptively transferred naïve 1-Derβ T cells proliferated in mice sensitised with HDM alone, but proliferated more in mice sensitised with HDM plus β-glucan (Fig. 1h). Moreover, in mice sensitised with HDM plus β-glucan, adoptively transferred 1-Derβ T cells expressed higher intracellular levels of the transcriptional factor GATA3 compared to 1-Derβ T cells from HDM sensitised mice or PBS controls (Fig. 1h). This enhanced Th2 polarisation of HDM-specific T cells could also be demonstrated by the increased levels of relevant cytokines, including IL-4, IL-5 and IL-13 that were produced by ex-vivo HDM stimulated MLNs (Fig. 1i). There was a slight increase in RORγT in 1-Derβ T cells from mice sensitised with HDM plus β-glucan, which did not translate into increased levels of IL-17 upon restimulation in vitro. There was no significant effect of β-glucan on IFN-γ production, but these carbohydrates did increase the production of IL-10 (Fig. 1i). Although we cannot exclude some contribution from innate lymphoid cells [15], we show here that particulate β-glucans exacerbate airway inflammation to HDM by promoting HDM-specific T cell priming.

## Abbreviations

AHR: Airway hyper-reactivity
HDM: House dust mite
IL: interleukin
PAMPs: Pathogen-associated molecular patterns
Th2: T helper 2
